# Development and Validation of a Measure to Assess Patient Experiences With Video Care Encounters

**DOI:** 10.1001/jamanetworkopen.2024.5277

**Published:** 2024-04-05

**Authors:** Cindie Slightam, Sonya SooHoo, Liberty Greene, Donna M. Zulman, Rachel Kimerling

**Affiliations:** 1 Center for Innovation to Implementation, Veterans Affairs Palo Alto Health Care System, Menlo Park, California; 2 Division of Primary Care and Population Health, Department of Medicine, Stanford University School of Medicine, Stanford, California; 3 National Center for PTSD, Veterans Affairs Palo Alto Health Care System, Menlo Park, California

## Abstract

**Importance:**

As video-based care expands in many clinical settings, assessing patient experiences with this care modality will help optimize health care quality, safety, and communication.

**Objective:**

To develop and assess the psychometric properties of the video visit user experience (VVUE) measure, a patient-reported measure of experiences with video-based technology.

**Design, Setting, and Participants:**

In this survey study, veterans completed a web-based, mail, or telephone survey about their use of Veterans Healthcare Administration (VHA) virtual care between September 2021 and January 2022. The survey was completed by patients who reported having a VHA video visit on their own device or a VHA-issued device and linked to VHA utilization data for the 6 months following the survey. Data analysis was performed from March 2022 to February 2023.

**Main Outcomes and Measures:**

The survey included 19 items about experiences with video visits that were rated using a 4-point Likert-type scale (strongly disagree to strongly agree). First, an exploratory factor analysis was conducted to determine the factor structure and parsimonious set of items, using the McDonald Omega test to assess internal consistency reliability. Then, a confirmatory factor analysis was conducted to test structural validity, and bivariate correlations between VVUE and VHA health care engagement were calculated to test concurrent validity. Finally, predictive validity was assessed using logistic regression to determine whether VVUE was associated with future VHA video visit use.

**Results:**

Among 1887 respondents included in the analyses, 83.2% (95% CI, 81.5%-84.8%) were male, 41.0% (95% CI, 38.8%-43.1%) were aged 65 years or older, and the majority had multiple chronic medical and mental health conditions. The exploratory factor analysis identified a 10-item single-factor VVUE measure (including questions about satisfaction, user-centeredness, technical quality, usefulness, and appropriateness), explaining 96% of the total variance, with acceptable internal consistency reliability (ω = 0.95). The confirmatory factor analysis results confirmed a single-factor solution (standardized root mean squared residual = 0.04). VVUE was positively associated with VHA health care engagement (ρ = 0.47; *P* < .001). Predictive validity models demonstrated that higher VVUE measure scores were associated with future use of video visits, where each 1-point increase on VVUE was associated with greater likelihood of having a video visit in subsequent 6 months (adjusted odds ratio, 1.04; 95% CI, 1.02-1.06).

**Conclusions and Relevance:**

The findings of this study of veterans using video visits suggest that a brief measure is valid to capture veterans’ experiences receiving VHA virtual care.

## Key Points

**Question:**

What measures should be used to assess patient experience with video visits?

**Findings:**

In this survey study of 1887 Veterans Affairs patients, a 10-item patient-reported measure was developed and validated to assess patient experience with video visits.

**Meaning:**

These findings suggest that the 10-item measure is a reliable and valid tool to evaluate patient experience with video visits.

## Teaser

This survey study assesses the psychometric properties of the video visit user experience measure, a patient-reported measure of experiences with video-based technology.

## Introduction

The rapid expansion of video-enabled telehealth has facilitated access to care^1^ but not without substantial implementation challenges, such as appropriate substitution for in-person care, given dynamic patient social and clinical needs, and a persistent digital divide.^2,3^ As telehealth policy and implementation strategies evolve to support sustained use of these technologies, monitoring patient experience with telehealth will be critical to foster meaningful use that has a positive impact on health care quality, access, and outcomes.^4^

Patient experience may be a uniquely important quality indicator to guide telehealth sustainment. Early studies of telehealth’s effectiveness suggest that video-based care largely offers quality comparable to that of in-person care, as indicated by standardized quality measures.^5^ The Consumer Assessment of Health Plans and Systems (CAHPS), a widely used and well-studied quality indicator for patient experience, has been shown to effectively capture common domains of experience across in-person and telehealth encounters, finding that patient experience is at least as positive for telehealth.^6^ The CAHPS has responded to COVID-19 pandemic–era increases in telehealth with the CAHPS Clinician and Group Visit Survey 4.0, which added a designation for visits by telephone and video visits and assessment of telehealth technical quality and ease of use.^7^

Burgeoning research on the patient experience with video visits suggests that additional domains of experience may be relevant for video visit quality improvement. For example, positive experiences are associated with better perceived usefulness and convenience, such as saving travel time and expense,^8,9,10^ or enhanced timeliness of care relative to in-person visits.^11,12^ Perceptions of appropriateness of the modality relative to in-person visits are important for positive experience with video visits, such as the acceptability of the lack of physical contact and/or examination, sufficient privacy for both patient and practitioner,^13,14,15^ and comfort interacting with the practitioner over video, such as being able to speak up and ask questions,^16^ also appear important for positive video visit experiences.^10,14,17^ More broadly, the degree to which video visits are perceived as user-centered, spanning both patient-centered interactions and the usability of technology, are factors associated with satisfaction with video visits and are associated with willingness to continue use of video visits.^10,18,19^ Research on overall satisfaction with video visits, including self-reported willingness to continue use of video visits, have found that it is an important indicator of future use.^20^

To date, few well-validated measures exist to guide the quality assurance for video-enabled telehealth services.^21^ Although good measures exist designed for implementation of specialty care, such as telepsychiatry services,^22^ none exist for widespread use across primary and specialty care video visits. A brief, pragmatic patient-reported measure of telehealth experience could help guide patient-centered implementation and sustainment of telehealth and facilitate rigorous, longitudinal studies of video-based care. The Veterans Healthcare Administration (VHA), a longstanding investor in telehealth, has made comprehensive efforts to implement, improve, and expand utilization of telehealth services (including video visits) to facilitate access to care, with rapid expansion during the COVID-19 pandemic.^23^ Several programs exist to promote access to video visits and bridge the digital divide, including making data services free of charge for health care use, and distribution of tablets to veterans who lack internet-enabled devices and broadband access.^23,24,25,26^ This makes the large nationwide VHA system an ideal environment to develop and test a video visit patient experience measure.

The objective of this study was to develop and psychometrically test a measure of video visit user experience (VVUE). We sought to create a brief, low-burden patient-reported measure that could express patients’ experiences with a single score. Measure development benefited from a national survey of VHA users that included candidate VVUE items, linked to administrative data to prospectively obtain video visit utilization. We established construct validity, reliability, and concurrent and predictive validity. We hypothesized that better VVUE scores would be associated with better health care engagement and with a greater likelihood of video visit utilization in the 6 months following the survey.

## Methods

### VVUE Item Generation

This project was designated as nonresearch by the Stanford University institutional review board and was conducted as a quality improvement evaluation in partnership with the VHA’s Office of Connected Care and Office of Rural Health. The project is reported according to the Strengthening the Reporting of Observational Studies in Epidemiology (STROBE) reporting guidelines for observational studies.^27^ Participants provided implied consent through the return of a completed survey.

To guide development of VVUE, we used a framework proposed before the COVID-19 pandemic,^28^ which we adapted into 5 content domains of user experience based on the subsequent video telehealth research. The study team developed an initial list of 27 candidate items drawn from the literature and grouped into our 5 content domains: satisfaction, user-centeredness, technical quality, usefulness, and appropriateness. To limit respondent burden, items were edited so that none exceeded an eighth grade reading level.

A panel of 7 VHA researchers, primary care clinicians, and psychologists (R.K.) with expertise in telehealth patient experience independently evaluated content validity of candidate items, rating each item on a 4-point Likert scale for relevance to the content domain and providing justification for ratings as free text. Items were retained (21 items) if they were rated as 3 or 4 (high relevance) by at least 4 experts. These items were then cognitively tested with up to 5 veterans using the think-aloud procedure followed by standardized verbal probes to elicit feedback on clarity, ease of comprehension, and relevance.^29^ Two items were removed, and 3 items were revised for clarity and retested. Ultimately, this process yielded 19 items across the 5 content domains (eTable 1 in Supplement 1).

### Participants and Procedures

**Figure 1.**
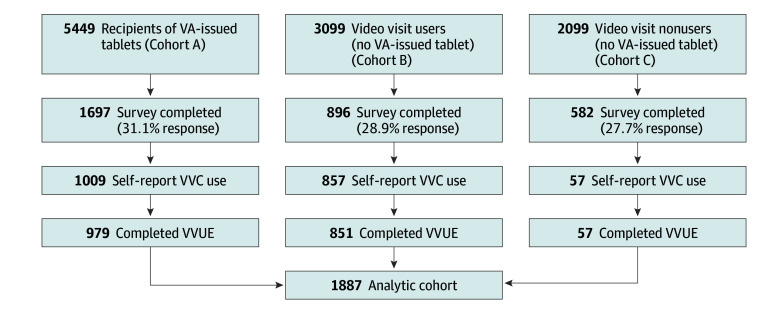
Survey Sampling and Response Flowchart Flowchart shows the sampling strategy that was used for the survey, including stratified sampling for 3 distinct cohorts: recent recipients of Veterans Affairs (VA)–issued tablets, non–tablet recipients who had a video visit documented in their health record, and non–tablet recipients who did not have a video visit documented in their health record. The analytic cohort is limited to survey respondents who reported having a video visit (self-report VA Video Connect [VVC] use) and completed the video visit user experience (VVUE) questions.

Quantitative psychometric data were collected as part of a survey that was conducted to assess access to and user experience of virtual care among VHA patients. This included veterans enrolled in a digital access program whereby those with clinical needs who lack the appropriate technology or infrastructure (devices, broadband, or internet access) can receive a video-enabled tablet and data plan for video-based care from home.^26,30^ A national multimode survey was linked to VHA administrative data to prospectively ascertain video visit utilization over the 6 months after the survey. Inclusion criteria were ascertained via administrative data and included VHA users aged 18 or older, with 2 or more primary care or mental health visits in the past year, and a valid US mailing address. Sampling was conducted as 3 cohorts: cohort A, recent (past 6 months) recipients of VHA-issued tablets distributed via the Office of Connected Care’s Digital Divide Consult^31^; cohort B, veterans with a video visit in the past 6 months who were not issued a tablet by VHA; and cohort C, veterans with no VHA video visit in the past year (Figure 1). Video visit use in the past 6 months was confirmed via survey self-report, and respondents from all cohorts who reported video visit use (eg, participants from the no video visit group who may have had a video visit after sample selection) were administered the candidate VVUE items. Because public-sector telehealth use varies by state,^32^ sampling was stratified by Veterans Integrated Services Networks to achieve geographic representation. Eligible veterans were emailed study information and a survey invitation with web, mail, or telephone survey options, and up to 2 email reminders. Nonresponders received a letter via US mail with the same information and 1 reminder postcard. Individuals who did not respond to the mailed survey were contacted by telephone by the survey contractor according to a protocol that specified a goal completion rate by the sample cohorts. Participants were compensated $10. Analyses for the current study included veterans with self-reported video visits within the past 6 months who completed all survey questions of interest (Figure 1).

### Data Sources and Measures

#### Survey Data

The 19 candidate items were administered in reference to the respondents’ most recent video visit. Response options were rated on a 4-point scale (strongly disagree to strongly agree). The Patient-Reported Outcomes Measurement Information System Healthcare Engagement Short Form, an 8-item patient reported measure that yields standardized *t* scores, was also administered via survey.^33^ Characteristics of the most recent video visit, gender, and race and ethnicity (categorized as African American, Hispanic/Latino, White, and other, which included American Indian or Alaska Native, Asian, Native Hawaiian/other Pacific Islander, other, and >1 race) were self-reported from survey respondents. Data on race and ethnicity were collected in an effort to include demographic and social variables that have demonstrated an association with video visit use in prior work.^14,26,34^

#### VHA Administrative Data

Age (date of birth), marital status, and geographic area (categorized as urban vs rural or highly rural, using 2010 US Census Bureau criteria based on home zip code^35^) were abstracted from administrative data. Chronic physical and mental health conditions were identified from *International Statistical Classification of Diseases and Related Health Problems, Tenth Revision* codes aggregated over past-year outpatient or inpatient encounters.^36,37,38^ Video visit outpatient utilization was aggregated over the 6 months following the survey and was identified using VHA Managerial Cost Accounting stop codes.

### Statistical Analysis

Data analysis was performed from March 2022 to February 2023. We examined item-level frequencies to assess skewness (±3), kurtosis (±10), and variation, ceiling, and floor effects and examined polychoric correlations to ensure items were at least moderately associated (*r *≥* *0.30) but not redundant (*r *≤* *0.80). Then, the Kaiser-Meyer-Olkin measure and the Bartlett test of sphericity were conducted to determine whether the data are adequate for factor analysis. We randomly split the sample to conduct exploratory factor analysis (EFA) models on one half to determine the factor structure, reserving the second half for confirmatory factor analysis (CFA). EFA was performed as a principal factor analysis of the polychoric correlation matrix with promax rotation. Models were fit iteratively, starting with a single factor model, to evaluate whether video visit experience is sufficiently unidimensional to be represented by a single scale score. EFA models were evaluated according to conceptual and empirical fit, including parallel analyses, scree plots, eignenvalues, and item loadings. Confirmatory models used weighted least-squared estimation^39^ and calculated the standardized root mean squared residual (SRMR) as an indicator of sufficient model fit (SRMR ≤0.08 indicates adequate fit).^40^ We calculated the McDonald Omega coefficient as an indicator of internal consistency.^41^ Item analyses and EFA were completed in Stata statistical software version 15 (StataCorp),^42^ and CFA was performed using Mplus statistical software version 8 (Muthén & Muthén).^43^

Concurrent validity was evaluated by the correlation between the VVUE item score and the Patient-Reported Outcomes Measurement Information System Healthcare Engagement Short Form.^33^ Predictive validity was examined using logistic regression to model the odds of 1 or more video visit among veterans with any outpatient health care utilization in the 6 months following the survey. Odds of video visit use were modeled as a function of VVUE scores, adjusting for patient characteristics that are associated with video visit utilization: age, race and ethnicity, sex, marital status, geographic area, and chronic physical and mental health conditions.^14,26,34^ We plotted marginal estimated probabilities over the continuum of VVUE scores to illustrate this association. All analyses were survey adjusted to account for stratified sampling. Statistical significance was set at 2-tailed *P* < .05.

## Results

### Participants

A total of 3178 veterans responded to the survey (29.8% response rate). The current sample is composed of the 1887 veterans who reported a video visit in the past 6 months and were administered the candidate VVUE items (Figure 1). Most respondents were male (83.2%; 95% CI, 81.5%-84.8%) and married (50.3%; 95% CI, 48.1%-52.5%), and 41.0% (95% CI, 38.8%-43.1%) were aged 65 years and older. More than one-half of the respondents reported their race as White (56.1%; 95% CI, 53.9%-58.2%), followed by African American (22.7%; 95% CI, 21.0%-24.5%), other (14.1%; 95% CI, 12.6%-15.7%), and Hispanic/Latino (6.9%; 95% CI, 5.9%-8.1%). More than one-quarter resided in a rural or highly rural area (27.9%; 95% CI, 26.0%-29.9%) (Table 1). In total, 41.1% (95% CI, 38.9%-43.2%) of respondents reported problems using video visits in the last 6 months, noting issues with connectivity (27.8%; 95% CI, 25.9%-29.8%), sound quality (19.0%; 95% CI, 17.3%-20.7%), video quality (19.2%; 95% CI, 17.5%-20.9%), lack of privacy (6.2%; 95% CI, 5.2%-7.4%), and lack of practitioner skills (5.9%; 95% CI, 4.9%-7.0%). Tablet recipients (cohort A) were more likely to report each of these problems than nontablet respondents (cohorts B and C) (eTable 2 in Supplement 1). Effect sizes for differences between survey respondents and nonrespondents were in the very small range, with the exception of older age among survey respondents in cohort B (standardized mean difference = 0.324) (eTable 3 in Supplement 1).

**Table 1.** Demographic and Clinical Characteristics of Survey Respondents^a^

Characteristic	Total VVUE analysis, % (95% CI) (N = 1887)
Age range, y^b^	
18 to <45	17.9 (16.3-19.7)
45 to <65	41.0 (38.8-43.1)
≥65	41.0 (38.8-43.1)
Marital status^b^	
Married	50.3 (48.1-52.5)
Separated, divorced, or widowed	33.5 (31.4-35.6)
Single or never married	16.1 (14.5-17.7)
Gender^c^	
Woman	15.7 (14.2-17.4)
Man	83.2 (81.5-84.8)
Nonbinary or other	1.0 (0.0-1.5)
Race and ethnicity^c^	
African American	22.7 (21.0-24.5)
Hispanic/Latino	6.9 (5.9-8.1)
White	56.1 (53.9-58.2)
Other^d^	14.1 (12.6-15.7)
Urban vs rural status^b^	
Highly rural or rural	27.9 (26.0-29.9)
Urban	72.0 (70.0-73.9)
No. of chronic physical conditions^b^	
<4	36.1 (34.0-38.2)
4-6	38.1 (36.0-40.2)
≥7	25.7 (23.9-27.6)
Any mental health condition^b^	
Yes	74.5 (72.5-76.3)
No	25.4 (23.6-27.4)
Patient reported VA video visit details^c^	
Type of device used for VA video visits^e^	
Smartphone	19.2 (17.5-20.8)
Tablet	3.4 (2.7-4.3)
Computer	12.7 (11.3-14.1)
VA-issued tablet	52.4 (51.2-53.4)
Other (eg, borrowed device)	0.7 (0.4-1.2)
More than 1 device reported	11.6 (10.3-13.0)
Most recent visit type	
Primary care	22.6 (20.8-24.4)
Mental health	48.3 (46.1-50.4)
Specialty care	12.9 (11.5-14.4)
Rehabilitation such as physical therapy, speech therapy	3.8 (3.0-4.7)
Not sure or other	12.3 (10.9-13.8)
Problems with VA video visits in past 6 mo	
Dropped connection	27.8 (25.9-29.8)
Poor sound quality	19.0 (17.3-20.7)
Poor video image quality	19.2 (17.5-20.9)
Lack of privacy (veteran location)	6.2 (5.2-7.4)
Lack of privacy (practitioner location)	3.7 (3.0-4.7)
Practitioner interrupted or distracted	3.9 (3.1-4.8)
Practitioner not skilled with video visits	5.9 (4.9-7.0)
Any of the above	41.1 (38.9-43.2)

Abbreviations: VA, Veterans Affairs; VVUE, Video Visit User Experience.

^a^ There were missing data in the overall sample for marital status (n = 20), gender (n = 7), and race (n = 23).

^b^ Data are from electronic health records.

^c^ Data are self-reported from survey.

^d^ Other includes American Indian or Alaska Native, Asian, Native Hawaiian/other Pacific Islander, other, and more than 1 race.

^e^ Type of device reflects the device(s) that tablet nonrecipients used for video visits (tablet recipients were not asked this question and are listed as using a VA-issued tablet).

### VVUE Development

Skewness, kurtosis, and variation were acceptable for all items. All interitem correlations exceeded 0.30, but several pairs or clusters of items were correlated greater than 0.80, suggesting redundant items. We removed 9 redundant items, retaining 1 item within each pair and/or cluster to preserve representation of each of the 5 content domains. The remaining 10 items fit a 1-factor solution, explaining 96% of the total variance and with all factor loadings greater than 0.70. The McDonald ω was 0.95, indicating adequate internal consistency reliability.

CFA confirmed unidimensionality, with factor loadings for all 10 items greater than 0.80 (Table 2) and an adequate fit to the data (SRMR = 0.04).^44^ Factor scores were highly correlated with summed scores (*r* = 0.98; *P* = .001), suggesting that summing VVUE items (strongly disagree = 1 to strongly agree = 4; composite scores 10-40) is appropriate. A higher VVUE score represents a more positive video visit experience (for final measure and scoring, see eTable 4 in Supplement 1).

**Table 2.** Factor Loadings From the CFA for the 10 Video Visit User Experience Items

Video visit user experience item	CFA factor loadings (n = 944)
“The video visit technology was easy to use”	0.83
“The video visit with the provider saved me time”	0.86
“There was enough technical support to help me complete the video visit”	0.84
“The lack of physical contact during the video visit was not a problem”	0.81
“The provider listened carefully to me during the video visit”	0.88
“The provider and I had the privacy we needed to complete the video visit”	0.87
“I received the same quality of care during the video visit compared to an in-person visit”	0.82
“I could see the provider clearly when he/she spoke to me”	0.88
“I could hear the provider clearly when he/she spoke to me”	0.90
“I would use video visits again”	0.88

Abbreviation: CFA, confirmatory factor analysis.

### Validity

**Figure 2.**
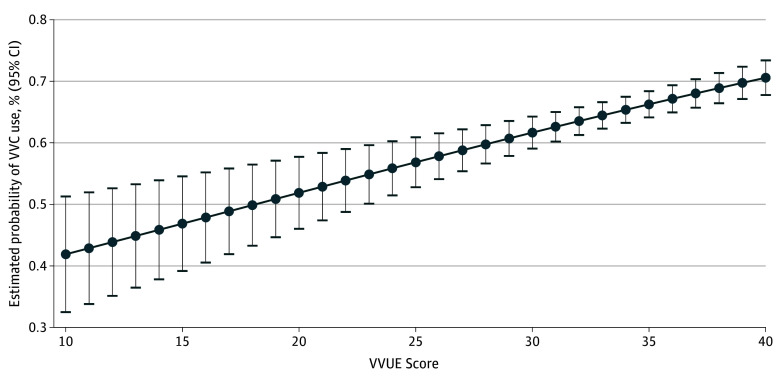
Adjusted Estimated Probabilities of VA Video Connect (VVC) Use Within 6 Months by Video Visit User Experience (VVUE) Score Graph shows the association of an individual’s VVUE score with the estimated probability of a video visit within 6 months, adjusted for age, race, marital status, gender, geographic area, number of physical health conditions, and any mental health conditions.

VVUE scores ranged from 10 to 40 (mean [SD], 33.8 [5.7]). There were no meaningful differences in mean (95% CI) VVUE scores by respondent demographic and clinical characteristics or by the device type (VA-issued or own device) (eTable 5 in Supplement 1). VVUE scores were significantly and negatively correlated with the number of problems reported with video visits (*r* = −0.37; *P* < .001), with mean (SD) VVUE scores of 35.3 (5.2) for those who reported no problems and 26.9 (6.3) for those who reported 4 or more problems. As expected, VVUE total scores were positively correlated with health care engagement scores (ρ = 0.47; *P* < .001), indicating good concurrent validity. Predictive validity models demonstrated that higher VVUE measure scores were associated with subsequent video visits utilization, where each 1-point increase on the VVUE was associated with a small but significant increase in the odds of a video visit within 6 months (adjusted odds ratio [AOR], 1.04; 95% CI, 1.02-1.06) (Table 3). Figure 2 illustrates the adjusted estimated probabilities of having a video visit within 6 months over the range of VVUE scores. When examined separately, we found VVUE remained significant among tablet recipients (AOR, 1.06; 95% CI, 1.03-1.08) and nonrecipients (AOR, 1.04; 95% CI, 1.01-1.06) (eTable 6 in Supplement 1).

**Table 3.** Association of VVUE Score With Future Video Visit

Variable	AOR (95% CI) (N = 1843)
VVUE score	1.04 (1.03-1.06)^a^
Age, y (reference, 18 to <45)	
45 to <65	1.10 (0.82-1.48)
≥65	0.87 (0.63-1.19)
Marital status (reference, married)	
Separated, divorced, or widowed	0.95 (0.76-1.18)
Single or never married	0.89 (0.66-1.20)
Gender (reference, woman)	
Man	1.00 (0.75-1.33)
Nonbinary or other	1.08 (0.42-2.77)
Race and ethnicity (reference, White)	
African American	1.03 (0.80-1.32)
Hispanic/Latino	0.93 (0.62-1.40)
Other^b^	0.91 (0.68-1.23)
Urban vs rural status (reference, rural)	
Urban	1.31 (1.05-1.62)^c^
Physical chronic conditions (reference, ≤3)	
4-6	1.28 (1.01-1.62)^c^
≥7	1.37 (1.04-1.79)^c^
Mental health condition (reference, no)	3.21 (2.57-4.02)^a^
Constant	0.14 (0.07-0.30)^a^

Abbreviations: AOR, adjusted odds ratio; VVUE, Video Visit User Experience.

^a^ *P* < .001.

^b^ Other includes American Indian or Alaska Native, Asian, Native Hawaiian/other Pacific Islander, other, and more than 1 race.

^c^ *P* < .05.

## Discussion

This survey study found that the VVUE is a reliable and brief, 10-item patient-reported measure that fills an important gap in patient-reported measures of virtual care experience. The VVUE is easy to score and poses a low burden to respondents, suggesting the measure is feasible for electronic or point-of-care administration. The content of the items addresses evidence-based characteristics of the video telehealth experience that underlie patient preferences and utilization of these services: satisfaction, user-centeredness, technical quality, usefulness, and appropriateness. The items in the questionnaire are also not VHA specific, and there is an opportunity to test this scale outside the VHA in other health care contexts.

Our sample included a substantial proportion of veterans who received VHA-issued tablets in addition to individuals who participated in video visits using their own devices, including smartphones, tablets, and computers. As a result, respondents were heterogeneous in terms of their access to internet connectivity and experience with video visits, broadening the representativeness of the sample. Consistent with patient experience data from other settings,^6,45^ veteran experiences were very positive. The majority of the sample reported no problems with video visits, resulting in high VVUE scores. The positive experiences may stem, in part, from VHA’s comprehensive approach to telehealth access and support, which predated the COVID-19 pandemic and spanned opportunities to improve access to video visits. For example, VHA has made substantial investments in telehealth training and support for staff and patients and has implemented initiatives to improve access to technology through the distribution of video-enabled devices and promotion of Federal Communications Commission programs that facilitate connectivity.^23,31^

A major benefit of video-enabled telehealth is its potential to promote continuity of care.^10,46^ Validity testing revealed that higher scores on the VVUE were associated with both better health care engagement and increased likelihood of continued video visit use in the 6 months after completing the VVUE. These findings are especially notable because VVUE scores demonstrated incremental validity to factors previously found to be associated with video visit utilization, including health status, the presence of chronic conditions, and demographic characteristics, such as race and ethnicity and age.^14,26,34^ These results suggest that the VVUE may provide useful information to guide the implementation and sustainment of telehealth services after the COVID-19 pandemic. These results also suggest that, among those with positive video telehealth experiences, the continued availability of these services may be an important component of patient-centered care.

### Limitations

Findings from these analyses should be interpreted in light of several caveats. The VVUE was developed through a survey of VHA patients. Although engagement of a public sector population with considerable health care and social need is a strength, the VHA is a large, integrated system with considerable uniformity in how telehealth is scheduled and accessed, and the results may not generalize to other settings. Future research is needed to determine effects of insurance status or issues such as language preferences and other factors that tend to be less salient for VHA populations.

## Conclusions

Video-enabled telehealth is increasingly a mainstay for delivery of medical and mental health care. This paradigm shift has transformed the way that patients interact with their health care team, leading to demand for patient-centered integration of telehealth services with traditional in-person care. The VVUE is a brief, reliable, and valid patient-reported measure of patient experience with video visits that places patient experience and preferences for telehealth at the center of implementation efforts.

## Notes

Supplement 1. **eTable 1.** Candidate Video Visit User Experience Items, by Content Domain **eTable 2.** Self-Reported Video Visit Problems by Device Type **eTable 3.** Comparison of Survey Respondents and Nonrespondents on EHR-Based Characteristics **eTable 4.** VVUE Measure and Scoring Instructions **eTable 5.** VVUE Score by Demographic and Clinical Characteristics **eTable 6.** Association Between VVUE Score and Future Video Visit by Device Type

Supplement 2.
Data Sharing Statement
