# Supplementary material for: Development and Validation of a Measure to Assess Patient Experiences With Video Care Encounters
Source: JAMA Netw Open. 2024 Apr 5;7(4):e245277. doi: 10.1001/jamanetworkopen.2024.5277 (PMC10998154; doi:10.1001/jamanetworkopen.2024.5277)
Supplement: Supplement 1. — eTable 1. Candidate Video Visit User Experience Items, by Content Domain eTable 2. Self-Reported Video Visit Problems by Device Type eTable 3. Comparison of Survey Respondents and Nonrespondents on EHR-Based Characteristics eTable 4. VVUE Measure and Scoring Instructions eTable 5. VVUE Score by Demographic and Clinical Characteristics eTable 6. Association Between VVUE Score and Future Video Visit by Device Type [file jamanetwopen-e245277-s001.pdf]

Supplemental Online Content

Slightam C, SooHoo S, Greene L, Zulman DM, Kimerling R. Development and validation of a measure to assess patient experiences with video care encounters. *JAMA Netw Open*. 2024;7(4):e245277. doi:10.1001/jamanetworkopen.2024.5277

eTable 1. Candidate Video Visit User Experience Items, by Content Domain

eTable 2. Self-Reported Video Visit Problems by Device Type

eTable 3. Comparison of Survey Respondents and Nonrespondents on EHR-Based Characteristics

eTable 4. VVUE Measure and Scoring Instructions

eTable 5. VVUE Score by Demographic and Clinical Characteristics

eTable 6. Association Between VVUE Score and Future Video Visit by Device Type

This supplemental material has been provided by the authors to give readers additional information about their work.

© 2024 Slightam C et al. *JAMA Network Open.*

eTable 1. Candidate Video Visit User Experience Items, by Content Domain

19 Video Visit User Experience Candidate Items	Domain
I would use video visits again*	Satisfaction
I was satisfied using video for the visit	
The provider listened carefully to me during the video visit*	User-Centeredness
Video visit technology was easy to use*	
The provider adequately addressed my health concerns during the video visit	
The provider included me in discussions about my care during the video visit	
There was enough time to talk to the provider during the video visit	
I felt confident about the quality of care I received during the video visit	
I felt comfortable using the video visit technology	
There was enough technical support to help me complete the video visit*	Technical Quality
I could see the provider clearly when he/she spoke to me*	
I could hear the provider clearly when he/she spoke to me*	
The video visit with the provider saved me time*	Usefulness
The video visit helped me get care in a timely manner	
The lack of physical contact during the video visit was not a problem*	Appropriateness
I received the same quality of care during the video visit compared to an in-person visit*	
The provider and I had the privacy we needed to complete the visit*	
I felt comfortable asking the provider questions about my health during the video visit	
The provider explained things in a way that was easy to understand during the video visit	

Note: *Indicates final VVUE Measure item

© 2024 Slightam C et al. *JAMA Network Open.*

eTable 2. Self-Reported Video Visit Problems by Device Type

Problems with VA video visits in past 6 months	Tablet Recipients (Cohort a) n=979	Own Device (Cohort b and c) n=908	p-value
Dropped connection	31.4 (28.5-34.2)	24.1 (21.3-26.9)	0.0003
Poor sound quality	20.7 (18.3-23.3)	17.2 (14.7-19.6)	0.04
Poor video image quality	21.1 (18.5-23.5)	17.2 (14.7-19.6)	0.03
Lack of privacy (Veteran location)	6.9 (5.4-8.5)	5.4 (3.9-6.9)	0.15
Lack of privacy (provider location)	4.7 (3.4-6.0)	2.8 (1.6-3.8)	0.02
Provider interrupted / distracted	4.3 (3.1-5.5)	3.4 (2.2-4.6)	0.31
Provider not skilled with video visits	7.1 (5.5-8.6)	4.6 (3.3-5.9)	0.02
Any of the above	44.9 (41.9-47.9)	36.9 (33.8-40.1)	0.0003

© 2024 Slightam C et al. *JAMA Network Open.*

eTable 3. Comparison of Survey Respondents and Nonrespondents on EHR-Based Characteristics

	Cohort A: VA-Issued Tablet Recipients			Cohort B: Other Video Visit Users			Cohort C: Video Visit Non-Users (No VA-Issued Tablet)		
	**Nonrespondent**	**Respondent**	**SMD**	**Nonrespondent**	**Respondent**	**SMD**	**Nonrespondent**	**Respondent**	**SMD**
n	3752	1697		2203	896		1517	582	
**Age**									
18-44	475 (12.7)	181 (10.7)	0.063	775 (35.2)	194 (21.7)	0.324	218 (14.4)	42 (7.2)	0.284
45 - 64	1384 (36.9)	635 (37.4)		779 (35.4)	337 (37.6)		435 (28.7)	139 (23.9)	
65+	1893 (50.5)	881 (51.9)		649 (29.5)	365 (40.7)		864 (57.0)	401 (68.9)	
**Gender**									
Female	413 (11.0)	236 (13.9)	0.088	376 (17.1)	138 (15.4)	0.045	120 (7.9)	41 (7.0)	0.033
Male	3339 (89.0)	1461 (86.1)		1827 (82.9)	758 (84.6)		1397 (92.1)	541 (93.0)	
**Race and Ethnicity**									
Black or African American	1151 (30.7)	473 (27.9)	0.069	504 (22.9)	156 (17.4)	0.215	261 (17.2)	79 (13.6)	0.19
Hispanic or Latino	205 (5.5)	104 (6.1)		244 (11.1)	69 (7.7)		81 (5.3)	23 (4.0)	
Other[1]	184 (4.9)	78 (4.6)		141 (6.4)	48 (5.4)		112 (7.4)	46 (7.9)	
Unknown/Missing	147 (3.9)	72 (4.2)		96 (4.4)	54 (6.0)		112 (7.4)	26 (4.5)	
White	2065 (55.0)	970 (57.2)		1218 (55.3)	569 (63.5)		951 (62.7)	408 (70.1)	
**Urban/Rural Status**									
Rural/Highly Rural	1122 (29.9)	518 (30.5)	0.027	525 (23.8)	233 (26.0)	0.083	562 (37.0)	223 (38.3)	0.057
Urban	2629 (70.1)	1179 (69.5)		1673 (75.9)	663 (74.0)		953 (62.8)	359 (61.7)	
Missing	1 (0.0)	0 (0.0)		5 (0.2)	0 (0.0)		2 (0.1)	0 (0.0)	
**Number Chronic Conditions** (Mean, SD)	7.51 (3.82)	7.45 (3.66)	0.017	5.57 (3.23)	5.76 (3.21)	0.058	4.32 (3.09)	4.35 (2.88)	0.01
**Any mental health condition**	3001 (80.0)	1330 (78.4)	0.04	1550 (70.4)	587 (65.5)	0.104	509 (33.6)	153 (26.3)	0.159
**Video visit in prior year**	2405 (64.1)	1300 (76.6)	0.276	—[2]					

[1] Includes American Indian or Alaska Native, Asian, Native Hawaiian or other Pacific Islander, More than one race.
[2] Based on the sampling strategy for the study survey, which assessed video visits in the prior year based on electronic health record data, all individuals in Cohort 2 had a video visit in the prior year, and none of the individuals in Cohort 3 had a video visit in the prior year.
Abbreviations: EHR Electronic Health Record; SMD Standardized Mean Difference.

© 2024 Slightam C et al. *JAMA Network Open.*

eTable 4. VVUE Measure and Scoring Instructions

Instructions: Please read the following statements and tell us about your experience with your most recent VA video visit.

	Strongly Disagree (1)	Disagree (2)	Agree (3)	Strongly Agree (4)
1. The video visit technology was easy to use	o	o	o	o
2. The video visit with the provider saved me time	o	o	o	o
3. There was enough technical support to help me complete the video visit	o	o	o	o
4. The lack of physical contact during the video visit was not a problem	o	o	o	o
5. The provider listened carefully to me during the video visit	o	o	o	o
6. The provider and I had the privacy we needed to complete the video visit	o	o	o	o
7. I received the same quality of care during the video visit compared to an in-person visit	o	o	o	o
8. I could see the provider clearly when he/she spoke to me	o	o	o	o
9. I could hear the provider clearly when he/she spoke to me	o	o	o	o
10. I would use video visits again	o	o	o	o

Scoring: Sum the 10 items to create a scale score (range of 10-40). Higher scores indicate a more positive experience with the video visit.

© 2024 Slightam C et al. *JAMA Network Open.*

eTable 5. VVUE Score by Demographic and Clinical Characteristics

	Total VVUE Sample (n=1,887)		VA-Issued Tablet Recipient (Cohort-a) (n=979)		Own Device (Cohort b and c) (n=908)	
	Mean and 95% Confidence Interval	p-value	Mean and 95% Confidence Interval	p-value	Mean and 95% Confidence Interval	p-value
**Age**						
18-<45	34.7 (34.2-35.3)	0.001	34.7 (33.8-35.6)	0.01	34.9 (34.1-35.6)	0.10
45-<65	33.8 (33.4-34.2)		33.7 (33.2-34.3)		34.1 (33.5-34.7)	
65+	33.4 (33.0-33.8)		33.2 (32.7-33.7)		33.8 (33.2-34.4)	
**Marital Status**						
Married	34.1 (33.7-34.4)	0.03	33.9 (33.3-34.4)	0.21	34.3 (33.8-34.8)	0.25
Separated/Divorced/Widowed	33.3 (32.9-33.8)		33.2 (32.6-33.8)		33.6 (32.9-34.3)	
Single/Never Married	34.0 (33.4-34.6)		33.9 (33.1-34.6)		34.4 (33.5-35.3)	
**Gender**						
woman	34.6 (33.9-35.2)	0.04	34.2 (33.3-35.2)	0.39	35.1 (34.2-36.0)	0.06
man	33.7 (33.4-34.0)		33.6 (33.2-33.9)		34.0 (33.6-34.4)	
nonbinary/other	33.0 (30.7-35.2)		32.8 (29.2-36.4)		33.2 (30.3-36.1)	
**Race/Ethnicity**						
African American	34.4 (33.9-34.9)	0.06	33.2 (32.7-33.7)	0.01	34.1 (33.6-34.6)	0.64
Hispanic/Latino	34.0 (33.0-35.0)		34.6 (34.0-35.2)		34.2 (33.4-35.1)	
White	33.6 (33.3-34.0)		33.2 (31.6-34.8)		34.9 (33.6-36.2)	
Other[1]	33.7 (33.0-34.4)		33.6 (32.5-34.6)		33.9 (33.0-34.9)	
**Urban/Rural Status**						
Highly rural/rural	33.8 (33.3-34.2)	0.80	33.3 (32.6-33.9)	0.19	34.5 (33.8-35.2)	0.28
Urban	33.8 (33.5-34.1)		33.8 (33.4-34.2)		34.0 (33.6-34.4)	
**Chronic Physical Conditions**						
<4	34.1 (33.7-34.5)	0.003	33.8 (33.3-34.4)	0.05	34.5 (33.9-35.0)	0.12
4-6	34.0 (33.6-34.4)		34.0 (33.5-34.6)		34.2 (33.6-34.8)	
7+	33.0 (32.5-33.6)		33.0 (32.3-33.6)		33.3 (32.4-34.3)	
**Any Mental Health Condition**						
Yes	33.7 (33.4-34.0)	0.25	33.8 (33.1-34.6)	0.57	34.2 (33.6-34.9)	0.65
No	34.1 (33.6-34.5)		33.6 (33.2-34.0)		34.1 (33.6-34.5)	

[1]Other includes the following categories: American Indian or Alaska Native, Asian, Native Hawaiian/other Pacific Islander, Other, and more than race.
Missing values: marital status (n=20), gender (n=7), race/ethnicity (n=23)

© 2024 Slightam C et al. *JAMA Network Open.*

eTable 6. Association Between VVUE Score and Future Video Visit by Device Type

	VA-Issued Tablet (n=956)		Own Device (n=887)	
	**OR**	**95% CI**	**OR**	**95% CI**
**VVUE score**	1.06***	[1.03,1.08]	1.04**	[1.01,1.06]
**Age** (ref: 18-<45)				
45-<65	0.92	[0.58,1.45]	1.13	[0.75,1.7]
65+	0.70	[0.43,1.13]	0.89	[0.58,1.38]
**Marital Status** (ref: married)				
Separated/Divorced/Widowed	0.94	[0.69,1.28]	0.87	[0.63,1.21]
Single/Never Married	0.92	[0.61,1.38]	0.72	[0.45,1.16]
**Gender** (Ref: woman)				
man	0.81	[0.53,1.24]	1.16	[0.77,1.75]
nonbinary/other	0.45	[0.14,1.43]	2.63	[0.55,12.73]
**Race/Ethnicity** (Ref: white)				
African American	0.74	[0.526,1.03]	1.36	[0.90,2.06]
Hispanic/Latino	1.05	[0.57,1.94]	0.80	[0.44,1.45]
other	0.75	[0.48,1.15]	0.99	[0.66,1.49]
**Urban/Rural Status** (ref: rural)				
urban	1.47*	[1.08,1.99]	1.25	[0.90,1.72]
**Physical Chronic Conditions** (Ref: <=3)				
4-6	1.25	[0.88,1.79]	1.21	[0.87,1.68]
7+	0.87	[0.60,1.27]	2.09***	[1.37,3.18]
**Mental Health Condition** (Ref=no)	2.72***	[1.89,3.91]	3.32***	[2.44,4.52]
Constant	0.22**	[0.08,0.64]	0.13***	[0.04,0.38]

© 2024 Slightam C et al. *JAMA Network Open.*
