# Supplementary material for: Development and Validation of a Measure to Assess Patient Experiences With Video Care Encounters
Source: JAMA Netw Open. 2024 Apr 5;7(4):e245277. doi: 10.1001/jamanetworkopen.2024.5277 (PMC10998154; doi:10.1001/jamanetworkopen.2024.5277)
Supplement: Supplement 2. — Data Sharing Statement [file jamanetwopen-e245277-s002.pdf]

Data Sharing Statement

Slightam. Development and Validation of a Measure to Assess Patient Experiences With Video Care Encounters. *JAMA Netw Open*. Published April 05, 2024.
doi:10.1001/jamanetworkopen.2024.5277

Data
Data available: No

Additional Information
Explanation for why data not available: Department of Veterans Affairs does not permit data sharing
